# Dynamic Disturbance Analysis of Grasslands Using Neural Networks and Spatiotemporal Indices Fusion on the Qinghai-Tibet Plateau

**DOI:** 10.3389/fpls.2021.760551

**Published:** 2022-01-17

**Authors:** Fengli Zou, Qingwu Hu, Haidong Li, Jie Lin, Yichuan Liu, Fulin Sun

**Affiliations:** ^1^School of Remote Sensing and Information Engineering, Wuhan University, Wuhan, China; ^2^Nanjing Institute of Environmental Sciences, Ministry of Ecology and Environment, Nanjing, China; ^3^Collaborative Innovation Center of Sustainable Forestry in Southern China of Jiangsu Province, Nanjing Forestry University, Nanjing, China; ^4^GeoScene Information Technology Co., Ltd., Beijing, China

**Keywords:** Qinghai-Tibetan Plateau, grassland, temporal-deep neural network classification, disturbance, change analysis

## Abstract

Grassland is the vegetation type with the widest coverage on the Qinghai-Tibet Plateau. Under the influence of multiple factors, such as global climate change and human activities, grassland is undergoing temporal and spatially different disturbances and changes, and they have a significant impact on the grassland ecosystem of the Qinghai-Tibet Plateau. Therefore, timely and dynamic monitoring of grassland disturbances and distinguishing the reasons for the changes are essential for ecological understanding and management. The purpose of this research is to propose a knowledge-based strategy to realize grassland dynamic distribution mapping and analysis of grassland disturbance changes in the region that are suitable for the Qinghai-Tibet Plateau. The purpose of this study is to propose an analysis algorithm that uses first annual mapping and then establishes temporal disturbance rules, which is applicable to the integrated exploration of disturbance changes in highland-type grasslands. The characteristic indexes of greenness and disturbance indices in the growing period were constructed and integrated with deep neural network learning to dynamically map the grassland for many years. The overall accuracy of grassland mapping was 94.11% and that of Kappa was 0.845. The results show that the area of grassland increased by 11.18% from 2001 to 2017. Then, the grassland disturbance change analysis method is proposed in monitoring the grassland distribution range, and it is found that the area of grassland with significant disturbance change accounts for 10.86% of the total area of the Qinghai-Tibet Plateau, and the disturbance changes are specifically divided into seven types. Among them, the type of degradation after disturbance mainly occurs in Tibet, whereas the main types of vegetation greenness increase in Qinghai and Gansu. At the same time, the study finds that climate change, altitude, and human grazing activities are the main factors affecting grassland disturbance changes in the Qinghai-Tibet Plateau, and there are spatial differences.

## Introduction

The dynamic change of vegetation cover is the main driving force of global environmental change (Avissar et al., 2002). Dynamic understanding and monitoring of the distribution of surface vegetation coverage is an important factor for environmental research (Schipper et al., 2014). The grassland ecosystem is one of the most widely distributed types of terrestrial ecosystems, covering about 37% of the earth’s terrestrial area (Piao et al., 2004; O’Mara, 2012) and playing an important role in the global ecosystem balance with ecosystem services, such as climate regulation, soil and water conservation, sand fixation, soil improvement, and biodiversity maintenance, especially in ecologically fragile plateau areas (Xie et al., 2008; Fan et al., 2012; Yue-Jun et al., 2012; Bernacchi and Vanloocke, 2015). Grassland is the most important vegetation type on the Qinghai-Tibet Plateau, accounting for 70% of the total vegetation area (Wang et al., 2013). Because of its excellent pasture quality, it is also one of the important grassland animal husbandry bases in China (Kemp et al., 2013). However, in recent years, under the dual effects of climate change and human activities, large-scale degradation of grassland on the Qinghai-Tibet Plateau has directly affected its ecological service functions. This situation restricts the sustainable development of alpine grassland animal husbandry, but the temporal and spatial dynamics of grassland degradation are not comprehensive and timely for environmental managers (David et al., 2008). Therefore, timely and accurate access to dynamic information on natural grassland is essential to realize the ecological sustainable development of the Qinghai-Tibet Plateau (Zavaleta et al., 2003).

In recent years, remote sensing has been widely used in regional and global vegetation cover monitoring. For the Qinghai-Tibet Plateau, where ground monitoring data is difficult to obtain, environmental monitoring combined with remote sensing image mapping has become more important (Xiao et al., 2009; Cornelius et al., 2013; Hansen et al., 2013). As a result, a large number of global land cover data sets exist, such as Global Land Cover 2000 (GLC2000) (Bartholome and Belward, 2005), MODIS’s Land Cover Collections (MAD12C5) (Friedl et al., 2010), and MERIS’s Global Cover (Arino et al., 2008), with resolution ranging from 300 m to 1 km. However, some studies also highlight the inconsistencies and uncertainties between these global land cover maps (Fritz et al., 2010; Tchuenté et al., 2011). The main problems are low accuracy and low levels of agreement between each other (Xiang et al., 2013). This makes it very inaccurate to obtain regional, high-precision, long-term dynamic grassland mapping based on global land cover data sets. We can improve land cover mapping based on remote sensing by using smarter algorithms, more suitable image acquisition time, and improved classification categories to make it more suitable for land cover research in characteristic areas (Tchuenté et al., 2011).

Research shows that a remote sensing index based on the time series of the year is an important method to effectively obtain vegetation phenological characteristics and can be used for land cover mapping and change monitoring (Cohen et al., 2010; Jamali et al., 2015; Ahmed et al., 2017; Hirschmugl et al., 2017). Methods of land use mapping and vegetation change detection based on remote sensing images are generally divided into two categories: machine learning algorithms and methods based on specific knowledge (Xie et al., 2008; Yin et al., 2020). With the development of remote sensing multisource big data, various machine learning algorithms are used more and more widely (Zhang et al., 2016; Qiu et al., 2017). However, in some areas where it is difficult to obtain sufficient training data and there is heterogeneity between different years of surface coverage, the superiority of machine learning algorithms is somewhat limited (Waldner et al., 2015; Shih et al., 2017). The method based on specific knowledge focuses on feature mining and geographic understanding, which can reduce dependence on a large amount of training data and overcome the drawbacks of machine learning algorithms to a certain extent. Knowledge-based methods mainly improve the detection and classification of remote sensing changes by constructing variables and proposing rules and hypotheses and are increasingly used for surface vegetation mapping and monitoring; however, these methods are limited by a fixed threshold of characteristic indicators (Mahyou et al., 2016; Qi et al., 2017). Therefore, the combination of prior knowledge, such as vegetation phenology, and machine learning models is one of the effective ways to improve land cover type mapping and change analysis based on remote sensing (Feng et al., 2021; Wang et al., 2021). However, long-term dynamic mapping and change analysis based on remote sensing images are a challenge for a large number of multisource data processing calculations (Mutanga and Kumar, 2019). Currently, free cloud platforms provide new methods for geospatial analysis. Google Earth Engine (GEE) avoids the preprocessing of large amounts of data, provides JavaScript and Python coding environment, facilitates data calculation, and improves the efficiency of application research based on remote sensing data (Liu et al., 2020).

So far, a large number of studies analyzes the grassland changes in the Qinghai-Tibet Plateau. Among them, the research based on remote sensing data is mainly based on the interannual trend changes of vegetation indicators, such as single NDVI and NPP, and the methods mainly include the NDVI trend threshold determination, trend residual, and vegetation net primary productivity evaluation methods (Genxu et al., 2004; Liang et al., 2007; Bian et al., 2008; Huilan and Yingjun, 2016; Chong-Yang et al., 2019). The study finds that climate change has a significant impact on the productivity of the entire Qinghai-Tibet Plateau (Zhang et al., 2007; Peng et al., 2012). In arid and semiarid regions, NDVI is related to rainfall and temperature and has strong spatial heterogeneity (Sun et al., 2016). In the source region of the Yangtze River, overgrazing causes serious grassland degradation, but at the same time (Li et al., 2013), some studies show that light intensity grazing has a positive effect on the productivity of alpine meadows (Zhang et al., 2015). These seemingly contradictory results indicate that the growth of grassland has a certain anti-disturbance ability to adapt to the environment, making the process of grassland change relatively complex and not a simple positive–negative trend change (Huang et al., 2016). Therefore, it is necessary to realize a more timely and comprehensive analysis of the grassland disturbance change process, especially in ecologically fragile plateau regions.

Based on the mapping of grassland dynamic distribution, this study comprehensively applies greenness and disturbance change indicators and further explores the analysis of the grassland disturbance change process in the Qinghai-Tibet Plateau without setting a subjective evaluation threshold. We aim to propose a comprehensive analysis algorithm that is applicable to the alpine cold region, which is first Annual Mapping and then establishes Temporal Disturbance Rules. Specifically (AMTDR). This study mainly includes three tasks: (1) to develop a novel grassland mapping algorithm by exploring the integration of the greening abundance index and disturbance index time-series characteristic deep neural network method that can be applied to the Qinghai-Tibet Plateau; (2) on the basis of dynamic mapping changes of grassland, to obtain multiyear grassland distribution maps and propose analysis rules for grassland disturbance changes in the Qinghai-Tibet Plateau from 2001 to 2017 with combining the trend changes of the greening abundance and disturbance indices; and (3) to explore the factors affecting the grassland disturbance changes in the Qinghai-Tibet Plateau from multiple perspectives, such as nature and humans.

## Materials and Methods

### Study Area

**FIGURE 1:**
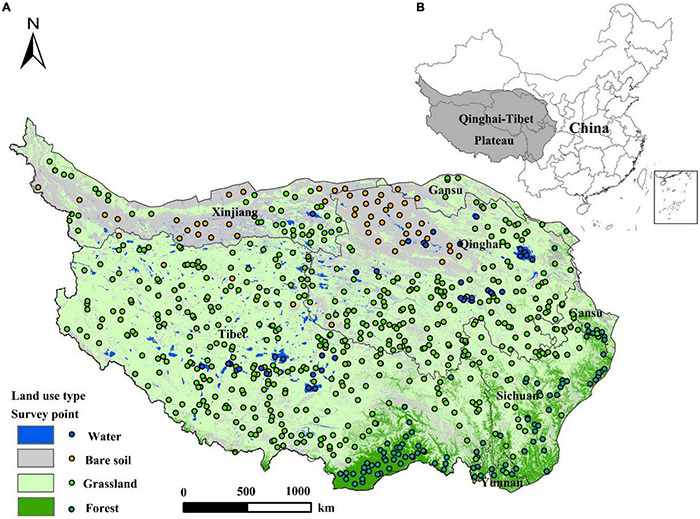
Land cover type and field survey points are located on the Qinghai-Tibet Plateau **(A)**. The location of the Qinghai-Tibet Plateau **(B)**.

The Qinghai-Tibet Plateau (26°50′–39°19′N, 78°25′–103°04′E) includes the Tibet Autonomous Region; Qinghai Province; and parts of Sichuan Province, Gansu Province, Xinjiang Uygur Autonomous Region, and Yunnan Province. With an average altitude above 4,000 m, it is known as the “roof of the world” and is the highest plateau with the highest average altitude in the world (Figure 1). In the unique alpine climate, from southeast to northwest, there are vegetation types, such as alpine meadow, alpine grassland, alpine semidesert, and alpine desert. Grassland resources are extremely rich, accounting for one third of the total grassland area in the country. It has been one of the important pastoral areas in China since ancient times. Among them, the eastern alpine meadow is not only the essence of grassland animal husbandry, but also the birthplace of rivers such as the Yellow and Yangtze Rivers. As a unique, ecologically sensitive, and fragile area in the world, the Qinghai-Tibet Plateau has a significant impact on global climate change and is also an important ecological barrier for China.

### Data Sources and Preparation

The MOD09A1 collection 6 data of the Qinghai-Tibet Plateau region from 2001 to 2017, synthesized for 8 days and with a spatial resolution of 500 m was used. The images use a quality layer (QA band) to exclude any 8-day composite images contaminated by clouds and perform geometric correction by pixel. The geo-reference datum is Mercator (UTM) projection and WGS 84 datum. In this paper, the linear interpolation method is used to encrypt the data and produce the daily data of 365 days a year.

Multiple spectral indices are calculated using MODIS time series data sets. Near infrared reflectance of terrestrial vegetation (NIRv) is the product of total scene NIR reflectance (NIR_*T*_) and the normalized difference vegetation index (NDVI). It can deal with the challenges of mixed pixels and the complexity of determining the photosynthetic capacity of vegetation (Badgley et al., 2017). Brightness, greenness, and wetness were derived based on the first, second, and third tasseled cap transformation (TC1 TC2 TC3) (Zhang et al., 2002).

Meteorological data were derived from the “grid data of temperature and precipitation over the QTP and surrounding areas from 2001 to 2017” released by the National Qinghai-Tibet Plateau Scientific Data Center^1^.

Reference points for the main land cover types of the Qinghai-Tibet Plateau are based on field surveys from July 2012 to September 2017 and visual interpretation of Google Earth’s high-resolution subimages: The total reference point is 696, of which grassland is 523, water is 24, bare soil is 53, and forest is 96, used for verifying the overall accuracy of grassland mapping (see locations in Figure 1).

Other data sets include stock census data and land cover data. Livestock census data is downloaded from the National Bureau of Statistics of China (NSBC)^2^, and they were collected through sampling statistics. The land use data used in this thesis include 2001, 2005, and 2010 data from the 30-m resolution GlobaLand30 product^3^ with an overall accuracy of 80.3% (Chen et al., 2015). Copernicus Global Land Cover CGLS-LC100 series 3 products 2015, 2017 data^4^ with overall accuracy at the continental level is around 80% with the highest accuracy of 83.7% for Asia (Buchhorn et al., 2020). GlobaLand30 and CGLS-LC100 series 3 land use data are better than the MODIS-MCD12Q1 land use product. In this paper, the land use data of 2001, 2005, 2010, 2015, and 2017 are resampled by 500. In the study area, 3,000 stable points without land use type change during 2001–2017 were randomly selected as the training data of the neural network deep learning model. Due to the particularity of the natural environment of the Qinghai-Tibet Plateau, the area suitable for crops only accounts for 0.9% of the area. Therefore, we aggregated the land cover map of 1:1 million land use survey results in 2001 into 500 m, which was used as the mask data to obtain the basic data containing only natural vegetation types. It is a natural land cover area and is further used for grassland identification and disturbance analysis.

### Methodology

**FIGURE 2:**
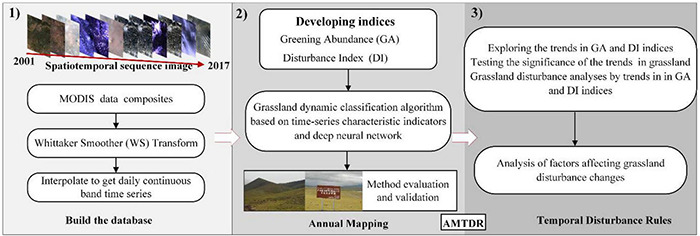
Methodological overview.

In this study, a comprehensive analysis algorithm is proposed for alpine regions by first AMTDR (Figure 2). Based on the fusion of knowledge-based time-series phenological features and neural network methods to achieve annual dynamic monitoring and mapping of grassland, the process of long time series grassland disturbance change is quantified and mined. It includes the following procedures: data preprocessing, construction of feature metrics, classification algorithms and validation, perturbation change rules, and influence factor analysis. The whole process is implemented based on Matlab, GEE^5^, and Python.

### Developing Indices of Greening Abundance and Disturbance

**FIGURE 3:**
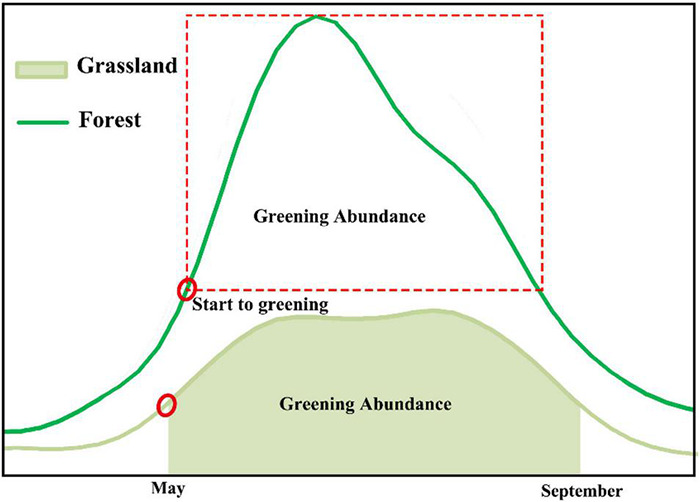
Time profile of annual growth knowledge-based features of NIRv grassland and forest in the Qinghai-Tibet Plateau region.

Due to the geographical characteristics of the alpine plateau in the Qinghai-Tibet Plateau, the annual phenology of all vegetation types in this area is one season, the main growth period is May to September (Zou et al., 2020), and the green coverage of different vegetation types during the growth period is relatively large (Figure 3). Therefore, this study obtains the greening abundance (GA) indicator by calculating the average value of the time profile between the maximum and minimum values of the NIRv indicator in the time series of the year from May to September.

Disturbance index (DI) is based on the linear combination of the three components of the tasseled cap transformation (Healey et al., 2005). It was originally used to detect forest disturbances, and then the algorithm was further adapted by looking for decadal changes in DI (Healey et al., 2005; Masek et al., 2008). In this study, we started with the original method (Healey et al., 2005) and calculated DI based on the standardized values (brightness, greenness, and wetness) of the tassel cap indicators, where the average and standard deviation of these indicators are based on a standardized data set adapted to the vegetation of the Qinghai-Tibet Plateau. The standard normalization formulas are as follows:


B=r(B-B)u/Bσ

G=r(G-G)u/Gσ

W=r(W-W)u/Wσ

Among them, *B*_*r*_, *G*_*r*_, and *W*_*r*_ are the standardized tasseled cap brightness, greenness, and wetness components. Therefore, the calculation formula of DI is:


DI=B-r(G+rW)r.

When the grass is disturbed, reducing the humidity and greenness and increasing the brightness make the DI value higher. For example, the grassland in the grazing area has a higher greenness and humidity at the beginning, and the surface is less exposed. When the grazing intensity increases rapidly, the humidity and greenness decrease, and the brightness increases significantly. If the grazing is controlled for a period of time thereafter, the greenness degree and humidity are restored to a certain extent. If the degree of grazing continues to increase, the greenness and humidity are further reduced.

**FIGURE 4:**
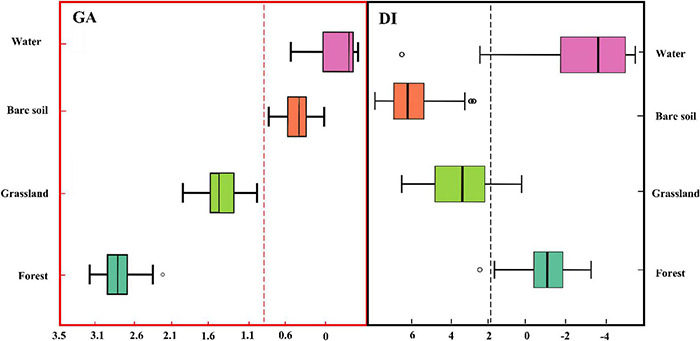
Different characteristics of natural land cover types on the Qinghai-Tibet Plateau in GA and DI indicators.

The main vegetation types on the Qinghai-Tibet Plateau are natural grassland and forest, of which grassland accounts for about 70% of the area of the Qinghai-Tibet Plateau, and other land covers are desert-type bare soil and water (Tian et al., 2014). In this study, we obtained 174 site locations for the four main types of land cover types, including grassland, forest, bare soil, and water, in the Qinghai-Tibet Plateau from field investigation and visual interpretation by Google Earth and obtained the time series of characteristics land types within the year that are stable on GA and DI indicators (Figure 4). When the vegetation is disturbed, increasing the brightness and reducing the greenness and wetness result in a higher DI value (Healey et al., 2005). Based on the GA calculated during the growing season, the greening abundance characteristics of different land cover types are further prominent.

### Grassland Classification

#### Deep Neural Network Model

Deep neural networks (DNN) are the foundation of deep learning. Neural networks are based on extensions of perceptrons, and DNN can be understood as neural networks with many hidden layers. DNN is also referred to as multilayer perceptron (MLP). According to the position of different layers of DNN, the neural network layer inside DNN can be divided into three types: input, hidden, and output layers. The layers are fully connected; that is, any neuron in layer 1 must be connected to any neuron in layer i +1. Although DNN looks very complex, for small local models, it is the same as a perceptron, that is, a linear relationship z = ∑*wixi + b* plus an activation function δ(z). Because there are many layers of DNN, our linear relationship coefficient W and the number of bias B also contain a lot. The neural network learns to represent the present state by some indicator. Then, take this index as the benchmark to find the optimal weight parameters. The neural network takes an index as a clue to find the optimal weight parameters. The index used in the learning of neural networks is called the loss function. This loss function can be represented by any function, and cross-entropy error is chosen in this paper. Cross-entropy error is also often used as a loss function. The loss can be described as


loss(x,class)=-log(exp(x[class])/∑jexp(x[j])=-x[class]log(∑exp(x[j]).

**FIGURE 5:**
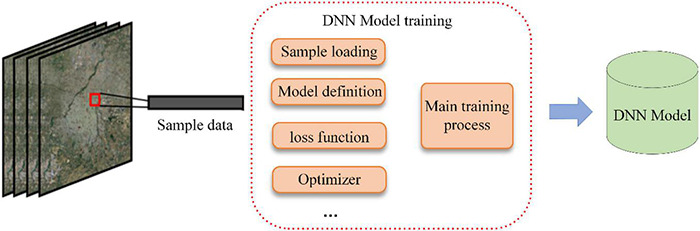
DNN model training process.

The process of the DNN model trained in this paper is as Figure 5. In this paper, a total of 3,000 sample data points were used for DNN model training, 80% of which were selected for model training and 20% for model accuracy verification. The accuracy of the model was 90.74%. Sample data for model training can be shared as accessible data through GEE.

#### Deep Neural Network Model Classification

**FIGURE 6:**
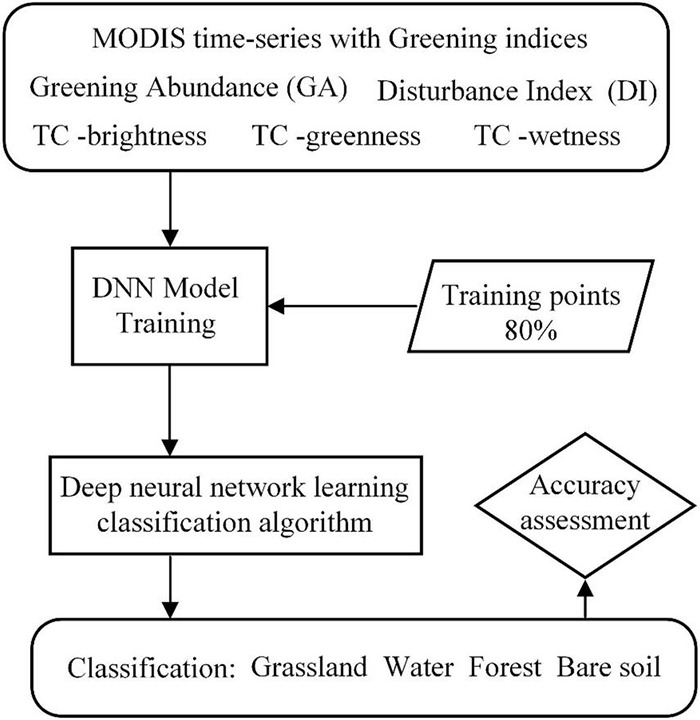
Flow chart of grassland classification.

The dynamic grassland mapping algorithm was developed by combining the characteristic indices representing the regional vegetation cover types on the Qinghai-Tibet Plateau with the deep network (Figure 6). The implementation process and part of pseudocode are as follows:

1. # Name the output training and test set

2. trainFilePrefix = ‘Training_data’

3. testFilePrefix = ‘Testing_demo’

4. # Training and testing data attributes (CLASS; Grassland, others (water body, forest land, bare soil), and contents of bands are 5*3 latitude attribute indexes

5. featureNames = list(bands)

6. featureNames.append(label)

7. trainingTask = image.select(bands).sampleRegions({

9. collection = training,

10. description = ‘ Training set ‘,

# Training DNN model in Pytorch

11. def DNN (trainDataset_dict, label)

12. DNNModel (trainDataset)

13. # Test the DNN model

14. model.evaluate(testDataset)

### Classification Algorithm Accuracy Verification

Accuracy assessment is based on reference sites. The sources of reference data are field surveys and high-resolution images of Google Earth. A random stratified sample design was used to evaluate the results of grassland recognition. A total of 696 reference points were collected, including one for grassland and one for other land cover types. The overall verification accuracy was 94.11%. Kappa was 0.845. And More specific verification information reference (Table 1).

**TABLE 1:** Accuracy assessment using ground reference sites.

	Total	Grassland	Other land cover types	Product accuracy (%)
Grassland	523	498	25	95.22
Other land cover types	173	16	157	90.75
User accuracy (%)		96.89	86.26	
Overall accuracy (%)	94.11			
Kappa	0.845			

### Grassland Disturbance Analysis Method

**FIGURE 7:**
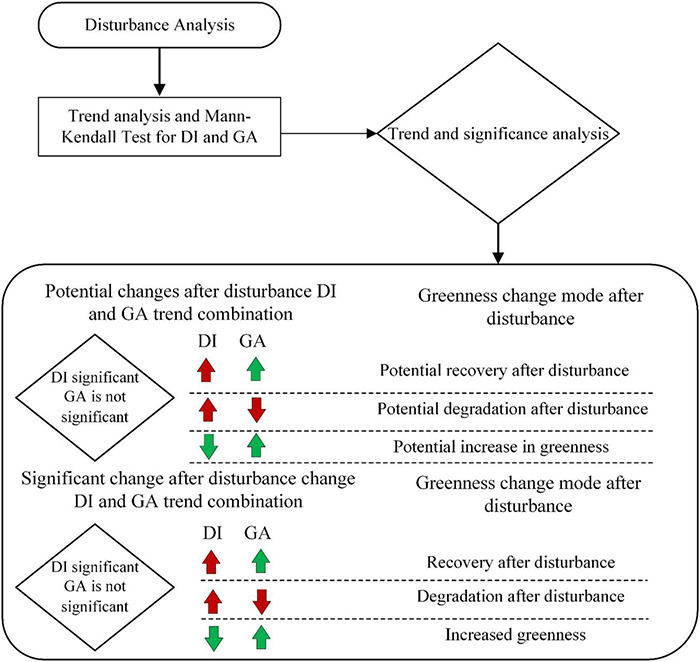
Establish analysis rules for grassland disturbance changes and corresponding change types in DI and GA indices.

When the grassland has a disturbance change, the DI value changes accordingly, so when the grassland has a disturbance change on an interannual basis, the DI shows an interannual trend change. When the grassland is disturbed to different degrees, its greening abundance shows different changes, corresponding to the trend of GA. To be able to analyze the interannual disturbance change form of grassland more accurately, we combined analysis of the possible change trend of DI and GA. The trend change range of the DI and GA indices is calculated according to the non-parametric Sen method. If the slope of Sen is obviously not equal to zero, there is a trend in the time series. At the same time, the MK test is used to quantify the degree of change in DI and GA index trends. When the DI trend is significant, it is quantified as a disturbance change. At the same time, when the corresponding GA trend is significant, it is quantified as a significant change in greenness after disturbance. When the trend of the GA indicator is not significant, it is quantified as the potential change of greenness after disturbance. The specific analysis rules are shown in Figure 7.

Because the growth of grassland has strong anti-interference, when the intensity of the disturbance of the grassland is within its anti-interference degree, although the green coverage changes in a trend, it does not change significantly. Define this potential change after disturbance: According to the positive and negative trends of green coverage, the types of potential changes can be divided into potential recovery after disturbance, potential degradation after disturbance, and potential greenness increase. When the intensity of the disturbance change in the grassland causes a significant trend change in the green coverage, this situation is judged as the type of postdisturbance change. According to the positive or negative trend of the green coverage, the significant change type can be divided into recovery after disturbance, degradation, and greenness increase after disturbance.

## Results

### The Temporal and Spatial Distribution Map of Grassland

**FIGURE 8:**
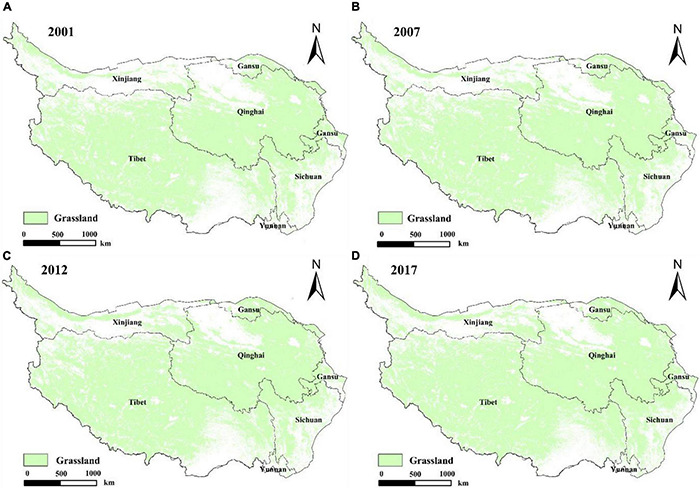
Grassland spatial distribution map from 2001 to 2017.

The grassland mapping algorithm based on GA and DI time series characteristic indices and deep network learning was applied to the multiyear grassland mapping, and the continuous dynamic distribution of grassland on the Qinghai-Tibet Plateau was obtained from 2001 to 2017 (Figure 8). The total area of the Qinghai-Tibet Plateau in the selected study area is 272.49 × 10^4^ km^2^. The grassland on the Qinghai-Tibet Plateau monitored by the DNN algorithm is mainly distributed in most areas of Qinghai and Tibet. The total area has increased from 165.25 × 104 km^2^ in 2001 to 174.34 × 104 km^2^ in 2007, 176.61 × 104 km^2^ in 2012, and 183.72 × 104 km^2^ in 2017; its spatial distribution is shown in Figures 8A–D; the overall area increases year by year and also fluctuates to a certain extent.

**FIGURE 9:**
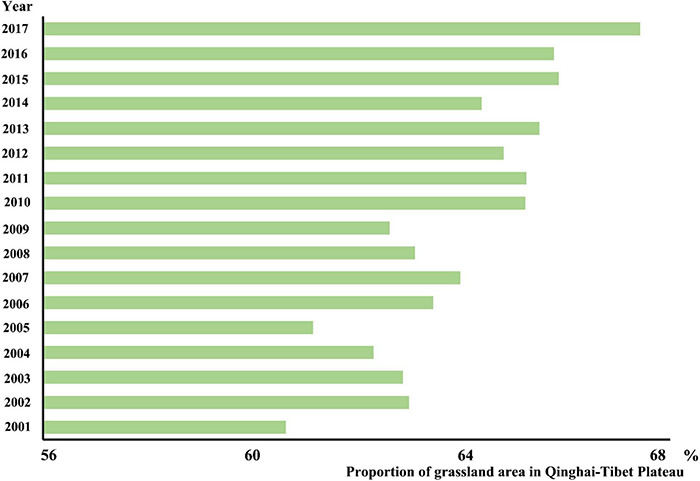
The percentage of grassland area in the Qinghai-Tibet Plateau area from 2001 to 2017.

From 2001 to 2017, the grassland area of the Qinghai-Tibet Plateau was greater than 60% of the entire region. From 60.65% in 2001 to 67.42% in 2017, the area increased by 11.18% in 17 years. Although the overall area is increasing, there are large fluctuations in each year, indicating that the grassland area is expanding, and there are also certain disturbances (Figure 9).

### Spatiotemporal Analysis of Grassland Disturbance Changes From 2001 to 2017

**FIGURE 10:**
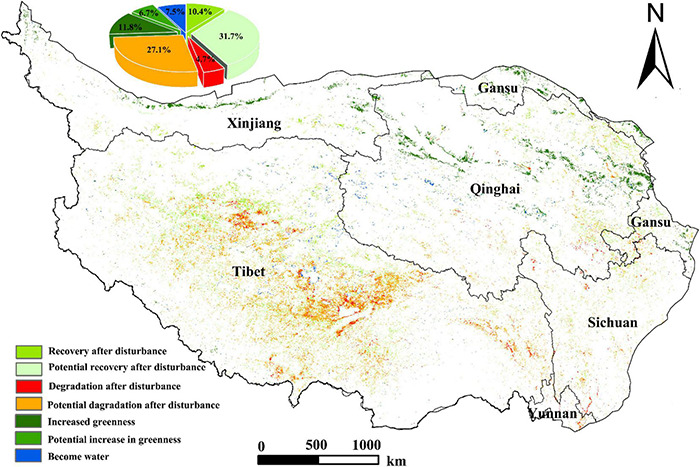
Distribution map of grassland disturbance change in the Qinghai-Tibet Plateau from 2001 to 2017.

According to the above disturbance analysis method, the distribution map of grassland disturbance changes in the Qinghai-Tibet Plateau region from 2001 to 2017 is obtained (Figure 10). It can be found that there are obvious spatial differences in the perturbation changes of grassland in 17 years. The regions with disturbance changes are mainly distributed in Tibet in the southwest of the Qinghai-Tibet Plateau, Qinghai in the northeast region and Gansu in the north. Among them, the types of degradation after the disturbance mainly occur in the Tibet region. In particular, the grasslands in central and southern Tibet are not only degraded in a large area, but also with a high degree of degradation. On the contrary, in Qinghai and Gansu, the main types of vegetation greenness increase are the main types. The vegetation coverage in this area is relatively sparse, and there are many desertification areas, but the grassland disturbance change type is a trend toward increasing greenness, indicating that, in this area, the ecological environment has improved to some extent in recent years. From 2001 to 2017, the grassland area in the study area with significant disturbance changes was 29.59 × 10^4^ km^2^, accounting for 10.86% of the total area of the Qinghai-Tibet Plateau. In the areas where there were disturbance changes, the recovery after disturbance accounted for 10.4%, mainly in the northern part of Tibet; the potential recovery after disturbance accounted for 31.7%, which is the largest change type, indicating that grassland has a certain potential for recovery from disturbance, mainly in the northern and central northern parts of Tibet. Degradation after disturbance accounted for 4.7%, mainly distributed in the southwestern part of the Qinghai-Tibet Plateau and central and northwestern parts of Tibet. Grassland in this area is significantly degraded and needs timely and effective attention and improvement; the potential degradation after disturbance accounts for 27.1%, which is also a large proportion. It is distributed in the southwestern part of the Qinghai-Tibet Plateau and most of Tibet. If these areas are effectively improved, there is still a certain possibility of recovery; the increased in greenness and the potential increase in greenness accounted for 11.8 and 6.7% respectively, mainly distributed in areas with relatively low vegetation coverage in the northern Qinghai-Tibet Plateau, such as Xinjiang, Qinghai, and Gansu. The area that turned into water accounted for 7.5%, mainly distributed on the edge of the lake distribution area in the middle of the Qinghai-Tibet Plateau, indicating that some lakes on the Qinghai-Tibet Plateau have a tendency to expand.

### Analysis of Influence Factors of Grassland Disturbance

#### The Impact of Climate on Grassland Disturbance

**FIGURE 11:**
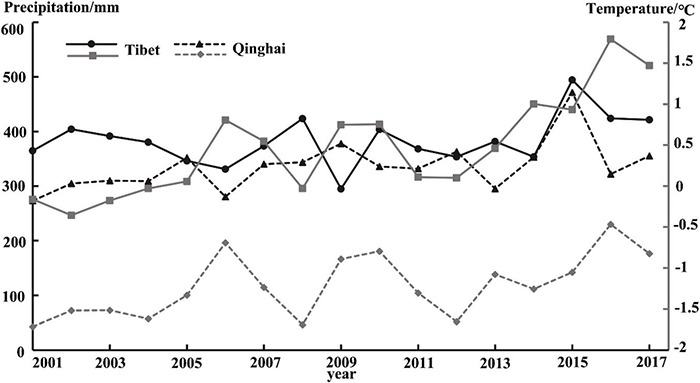
Changes in temperature and precipitation in Qinghai and Tibetan from 2001 to 2017.

Due to the special geographical environment of the Qinghai-Tibet Plateau, the climate in the region has strong spatial heterogeneity. According to the above analysis (Figure 9), it can be found that the areas of grassland disturbance change in the Qinghai-Tibet Plateau are mainly distributed in Tibet and Qinghai provinces. Therefore, we take the main regions of Qinghai and Tibet as examples to calculate the changes in climate trends from 2001 to 2017 (Figure 11) and do a correlation analysis with the DI change trend of the corresponding grassland. In terms of precipitation, the precipitation in Tibet is in the range of 294.88∼494.23 mm with an average of 382.95 mm; the precipitation in Qinghai Province is in the range of 273.11∼471.6 mm with an average of 336.35 mm. In terms of temperature, the temperature in Tibet is in the range of −0.36∼1.79°C with an average of 0.47°C. The temperature in Qinghai Province is in the range of −1.71∼−0.47 with an average of −1.21°C. From 2001 to 2017, the temperature and precipitation in Tibet and Qinghai show an overall upward trend. The correlation coefficient between precipitation trend and grassland disturbance change trend in Tibet is 0.33, and the correlation coefficient between precipitation trend and grassland disturbance change trend in Qinghai is 0.122; the correlation is less than 0.5, indicating that the precipitation and grassland disturbance change have a certain correlation but the impact is small. The correlation between temperature and the grassland disturbance change trend in Tibet is 0.66, and the change trend of temperature and grassland disturbance in Qinghai Province is 0.23. The correlation between temperature in Tibet and Qinghai on grassland disturbance is higher than precipitation, especially in Tibet, which is located in the southern Qinghai-Tibet Plateau. All in all, in the main areas of the Qinghai-Tibet Plateau, climate is one of the factors affecting grassland disturbance changes, and there is spatial heterogeneity. In the southern part of the Qinghai-Tibet Plateau, temperature has a greater impact on the disturbance of grassland, whereas precipitation has a small impact on the disturbance of grassland. In the northern part of the Qinghai-Tibet Plateau, both temperature and precipitation have a small impact on the disturbance and change of grassland, but the same is the greater impact of temperature. Therefore, temperature is the main factor affecting grassland disturbance changes among climate factors, and it has a greater impact on the southern part of the Qinghai-Tibet Plateau. The rising trend of temperature and precipitation in the northern Qinghai-Tibet Plateau is one of the main reasons for the increase in grassland greenness. However, the types of grassland changes in the southern Qinghai-Tibet Plateau after disturbance are complex. Climate factors are not the only factors affecting grassland disturbance changes, and further exploration and analysis are needed.

#### Influence of Elevation on Grassland Disturbance

**FIGURE 12:**
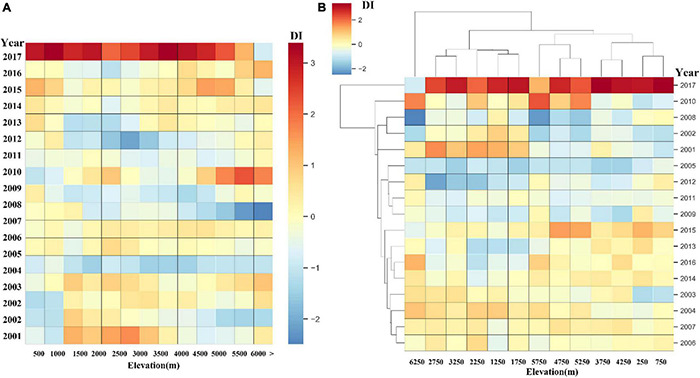
The annual average DI values of grassland with elevation in different altitudinal zones from 2001 to 2017.

As the altitude changes, the vertical spatial distribution and distributed clustering of the grassland DI index in the Qinghai-Tibet Plateau is shown in Figures 12A,B. Combining A and B in Figure 12, we can find that the DI value of grassland increases with different heights of sea waves, and the altitude varies from < 1,000 to > 6,000, showing different trends. When the altitude is less than 1,000 m, the trend of grassland disturbance index decreases with the increase of altitude, and the value of each year fluctuates slightly. When the altitude is between 1,000 and 2,500 m, the trend of grassland disturbance index increases with the increase in altitude, and the fluctuation of each year begins to increase. When the altitude is greater than 3,000 m, the change trend of the disturbance change index of the grassland presents an unstable fluctuating change that first decreases, then rises, and then decreases, and when the altitude is higher than 4,500 m, the DI value fluctuates in different years at the same altitude and is also larger. It can be found that the change trend of grassland disturbance on the Qinghai-Tibet Plateau is dependent on altitude, but it is not a simple linear relationship. Among different altitudes, the correlation between grassland disturbance changes and low altitude is greater than that of high altitude. In the same altitude, the higher the altitude, the greater the fluctuation trend of grassland disturbance changes in each year. The study also found that in 2005–2010, 2010–2015, and 2015–2017, the degree of grassland disturbance gradually increased when the altitude was in the range of 3,000–6,000 m, and the relationship between grassland disturbance and altitude was small when the altitude was below 3,000 m.

#### Influence of Human Grazing on Grassland Disturbance

**FIGURE 13:**
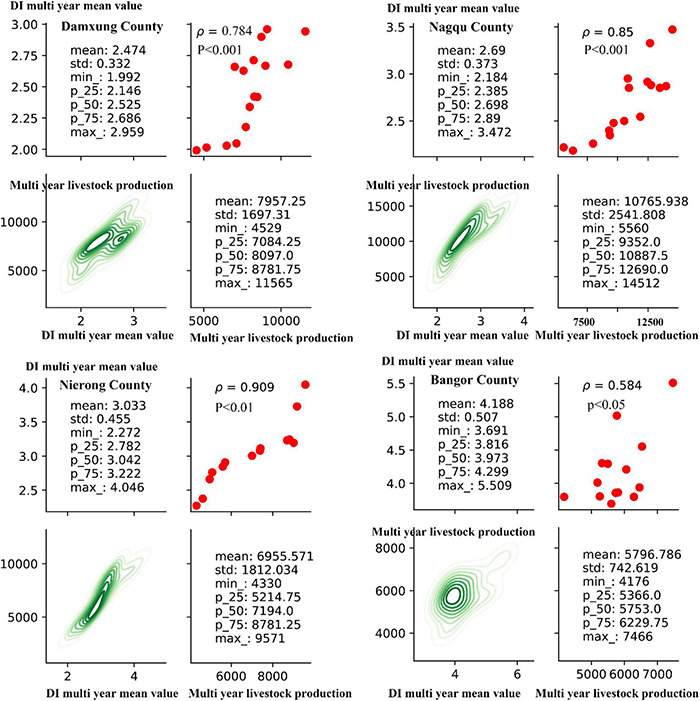
Correlation between the annual average value of grassland DI in the main distribution areas of grassland disturbance degradation types on the Qinghai-Tibet Plateau and the corresponding annual livestock production.

Grazing is one of the most important uses of grassland ecosystems in the Qinghai-Tibet Plateau. Overgrazing may be an important reason for grassland degradation (Hong-Qin et al., 2018). From the above analysis of grassland disturbance changes, it can be seen that the main area of greenness degradation after grassland disturbance is central and southern Tibet, and the main geographical areas of its specific distribution are Damxung, Nagqu, Nierong, and Bangor Counties. In this area, the interannual trend changes of the grassland DI index from 2001 to 2017 and the correlation analysis with the interannual change trend of the annual livestock production statistics in the corresponding area are shown in Figure 13. It can be found that, in the corresponding areas in which the greenness of the grassland is significantly degraded after the disturbance, the DI index in 2001–2017 showed a significant upward trend. It can be found that, in the corresponding areas where the greenness of the grassland is significantly degraded after the disturbance, the DI index in 2001–2017 showed a significant upward trend, and the correlation coefficients with the corresponding interannual variation trend of livestock production were all greater than 0.5, of which Damxung County in the regions of Nagqu and Nierong Counties, the correlation coefficients were 0.78, 0.84, and 0.91, respectively, with *p* < 0.01, showing a very significant positive correlation. The correlation coefficient in the Bangor County area is 0.58, *P* < 0.05, which is a significant positive correlation. It can be proved that, in areas where the greenness of the grassland is degraded after disturbance, the upward trend of livestock production is the same as the change trend of grassland disturbance, and the correlation is high. Therefore, grazing is one of the main causes of disturbance degradation in the greenness degraded area of the Qinghai-Tibet Plateau grassland after disturbance. In addition, from the characteristic values found in the graph, the eigenvalues (mean, STD, Max, min, percentile_value) of the DI multiyear changes in different areas and the change of multiyear livestock production in the area of characteristic value was no regular distribution because of the different quality of grass growth in different regions. More data support and more in-depth research are needed to obtain the boundary carrying value of livestock quantity that does not affect grassland growth change. Reasonable grazing planning in areas degraded by grassland disturbances is worthy of attention to regional ecological management.

## Discussion

As a unique natural area on the earth, the Qinghai-Tibet Plateau is a sensitive area and ecologically fragile zone of global climate change, and grassland is the most important ecological security barrier in the region (Yuan-He and Shi-Long, 2006). How to dynamically monitor the spatiotemporal changes of grassland and realize the exploration and excavation of its long-term change process mechanism has become an urgent need. In the evaluation study of grassland degradation grades based on vegetation coverage and empirical thresholds, as of 2013, the grassland degradation area of the Qinghai-Tibet Plateau accounted for about 30% with slight degradation as the main component (Xu-juan et al., 2019). Based on the algorithm of AMTDR, this study uses mathematical statistics to obtain 10.86% of the total area of the Qinghai-Tibet Plateau that has undergone significant disturbance changes as of 2017. The main type of change is recovery after disturbance. This comparison shows that the restoration effect of degraded grassland in the Qinghai-Tibet Plateau has been significant in recent years, which is similar to the results of some related policies and research findings in the region. For example, the national and local governments have successively carried out grassland ecological protection projects in the Three River Sources of Qinghai and Qiangtang in Tibet, which have obvious effects on the restoration of grassland degradation (Xu et al., 2017). In the Three River Source area of Qinghai, nature reserves and national parks have been established successively, and a series of measures have been implemented, such as retiring people, reducing livestock, and returning grazing to grassland, and the ecological environment has been significantly improved (Shao et al., 2016). Based on remote sensing data, the more specific disturbance changes of grassland can be obtained comprehensively and objectively under the introduction of non-parameters. To a certain extent, this overcomes the existing problems of grassland degradation research in the Qinghai-Tibet Plateau region: In the case of difficulty in obtaining field monitoring data, there is a lack of a deeper dynamic quantitative monitoring of the grassland disturbance change process in the region.

Grassland disturbance changes are the result of the combined effects of many factors. Climate change and human activities have an important impact on the grassland growth process, but the impact trend is not necessary (Cui and Graf, 2009; Cai et al., 2014). Regionally, Qinghai and Tibet have different water vapor sources and heating systems (Yang et al., 2014). The altitude of Tibet is relatively higher than Qinghai, which makes Tibet’s temperature warming trend faster, and the relatively higher vegetation coverage in the Qinghai area can change the surface sensible heat flux through stronger evapotranspiration to slow the surface warming effect (Jeong et al., 2009). This is consistent with the results of the study in this study that the temperature in southern Qinghai-Tibet Plateau, which is dominated by Tibet, has a higher impact on grassland disturbance changes than that in Qinghai, which is dominated by the northern Qinghai-Tibet Plateau. The spatial pattern of precipitation is more complicated than temperature changes (Zhang et al., 2013). In this study, the linear correlation of the influence of precipitation on grassland disturbance changes in the southern and northern Qinghai-Tibet Plateau is not significant.

Because Tibet in the southern part of the Qinghai-Tibet Plateau is more unfavorable to economic development than Qinghai in the northern part of the Qinghai-Tibet Plateau, the environmental factors are more unfavorable, and the economic structure of Tibet is relatively single, which makes Tibet more dependent on the development of animal husbandry (Huang et al., 2016). In recent decades, the rapid development of Tibet’s increased efforts in animal husbandry has made Tibet’s total livestock grazing increase faster than Qinghai. In this study, in the areas of grassland disturbance change, the areas where grassland degradation occurred were mainly concentrated in southern Tibet in the southern part of the Qinghai-Tibet Plateau, and the main reason for the degradation was overgrazing.

The accuracy of this research is affected by some facts. Although the process of grassland disturbance change can be further dynamically quantified and monitored, it cannot directly indicate the cause and effect of the change. In this paper, due to the limitation of the spatially explicit data of the regional grazing intensity, the analysis of the quantified process of the disturbance change and the reasons for the disturbance change are not in-depth enough. In addition, the inconsistency of the hysteresis effects of climate change and overgrazing requires the use of comprehensive models supported by more relevant factors to further explore the driving mechanism.

## Conclusion

As the main distribution region of alpine grassland in the world, the Qinghai-Tibet Plateau is sensitive to climate change, so it is very important to grasp the spatiotemporal pattern of the grassland ecosystem in time to predict the ecological environment change safely. This research relies on a large number of remote sensing application data sets, and the GEE platform is combined to solve the problems of data acquisition, processing, and storage. We propose a kind of suitable for comprehensive analysis algorithm in high latitude area, the first AMTDR. In this method, based on the combination of knowledge-based, time-series phenology characteristics and DNN method to achieve the multiyear dynamic monitoring mapping of grassland, the multiyear time-series characteristics of feature indexes were further explored, and the rule algorithm for analyzing the changes of temporal disturbances of grassland was proposed, which is independent of the index threshold division. Therefore, it has realized the comprehensive continuous dynamic monitoring of multiyear disturbance change mode by monitoring the spatiotemporal mapping of alpine grassland year by year in a large area and long time series and further explored the application of remote sensing image in the monitoring of alpine grassland ecosystem change.

Based on the long-term monitoring of grassland disturbance changes, the relationship between climate change, altitude and human grazing activities and grassland disturbance changes in the Qinghai-Tibet Plateau is also briefly quantified. In this study, in the main areas of the Qinghai-Tibet Plateau, climate is one of the factors affecting grassland disturbance changes, and there are differences in spatial distribution. The influence on the southern part of the Qinghai-Tibet Plateau is greater than the northern part, but in the northern part of the Qinghai-Tibet Plateau, temperature is one of the main reasons for the increase in grassland greenness. The temperature in the southern part of the Qinghai-Tibet Plateau is just one of the reasons for the various disturbances in the grassland. The grassland disturbance change trend of the Qinghai-Tibet Plateau has a relationship with altitude. Among different altitudes, the correlation between grassland disturbance changes and low altitude is greater than that of high altitude. At the same altitude, the higher the altitude, the greater the annual fluctuation trend of grassland disturbance. In areas where grassland greenness is degraded after disturbance, the increasing trend of livestock production is the same as that of grassland disturbance, and the correlation is high. Therefore, grazing is one of the main causes of disturbance degradation in the green degradation area of Qinghai-Tibet Plateau grassland after disturbance.

## Data Availability Statement

The raw data supporting the conclusions of this article will be made available by the authors, without undue reservation.

## Author Contributions

FZ: conceptualization, methodology, software, data curation, and writing—original draft preparation. HL and JL: visualization. QH, YL, and FS: investigation and data curation.

## Conflict of Interest

FS was employed by the company GeoScene Information Technology Co., Ltd. The remaining authors declare that the research was conducted in the absence of any commercial or financial relationships that could be construed as a potential conflict of interest.

## Publisher’s Note

All claims expressed in this article are solely those of the authors and do not necessarily represent those of their affiliated organizations, or those of the publisher, the editors and the reviewers. Any product that may be evaluated in this article, or claim that may be made by its manufacturer, is not guaranteed or endorsed by the publisher.

## Footnotes

1
http://www.tpdc.ac.cn/zh-hans/data/c954daad-6086-4edd-a6c5-f69c581e5c31/

2
http://www.stats.gov.cn/english/

3
http://www.globallandcover.com/

4
https://developers.google.com/earth-engine/datasets/catalog/COPERNICUS_Landcover_100m_Proba-V-C3_Global?hl=en

5
https://earthengine.google.com
