# Supplementary material for: Dynamic Disturbance Analysis of Grasslands Using Neural Networks and Spatiotemporal Indices Fusion on the Qinghai-Tibet Plateau
Source: Front Plant Sci. 2022 Jan 17;12:760551. doi: 10.3389/fpls.2021.760551 (PMC8801810; doi:10.3389/fpls.2021.760551)
Supplement: Supplementary file 1 [file Data_Sheet_1.PDF]

Additional description for DNN model

- Further explanation of the mechanism and applicability of DNN method model

DNN is essentially an MLP model. An MLP consists of three main layers of nodes — an input layer, a hidden layer, and an output layer. In the hidden and the output layer, every node is considered as a neuron that uses a nonlinear activation function. Nodes are little parts of the system, and they are like neurons of the human brain. When a stimulus hits them, a process takes place in these nodes. Some of them are connected and marked, and some are not, but in general, nodes are grouped into layers. A deep neural network is beneficial when you need to replace human labor with autonomous work without compromising its efficiency. In order to improve the applicability of the model, we simplified the structure of the model based on the interrelationships among the feature indicators evaluated by the experimental points. According to repeated experiments, we have only two hidden layers in addition to the input and output layers in the model design, and the number of channels in the hidden layer is dynamically adjusted as a hyperparameter.

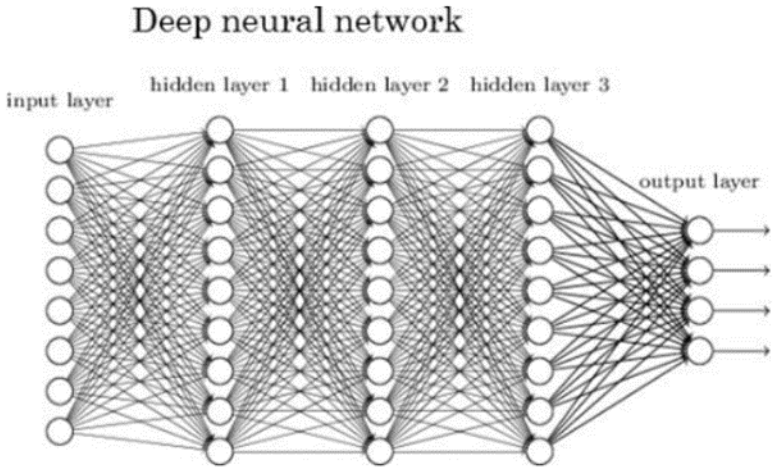

Figure1: DNN model.

- How to improve the accuracy of the model, solve the over-fitting problem and impr ove the generalization of the model

We take the average value of the five characteristic indicators selected in this experiment to give an evaluation example. (In the specific experiment, in addition to the average value, it also includes the maximum value of each indicator. The elevation data is the empirical auxiliary value in the remote sensing image classification)

1. Simplify the model structure:

We first performed exploratory data analysis on more than 3,000 samples, and obtained the ratio of positive samples to negative samples: 1830:1669, which shows that there is no imbalance problem in the samples. Then do a correlation analysis on the 7 indicators (the results are shown in the figure2). From the analysis results, it can be seen that the geeen and va features have higher correlation than other features. In view of the large difference in correlation between features, the more complex the linear model, the easier it is to overfit. Therefore, in the model design, apart from

the input and output layers, there are only two hidden layers (as shown in Figure 3), and the number of channels in the hidden layer is dynamically adjusted as a hyperparameter to obtain the optimal result through trial and error.

	ELEVATION	SLOPE	bright	di	geeen	va	wet	class
ELEVATION	1.00	-0.07	0.28	0.05	-0.39	-0.45	0.19	0.11
SLOPE	-0.07	1.00	-0.19	-0.47	0.18	0.33	0.32	-0.00
bright	0.28	-0.19	1.00	0.44	-0.61	-0.49	0.09	-0.22
di	0.05	-0.47	0.44	1.00	-0.27	-0.41	-0.79	-0.03
geeen	-0.39	0.18	-0.61	-0.27	1.00	0.92	-0.26	0.47
va	-0.45	0.33	-0.49	-0.41	0.92	1.00	-0.05	0.37
wet	0.19	0.32	0.09	-0.79	-0.26	-0.05	1.00	-0.20
class	0.11	-0.00	-0.22	-0.03	0.47	0.37	-0.20	1.00

Figure2： Correlation analysis.

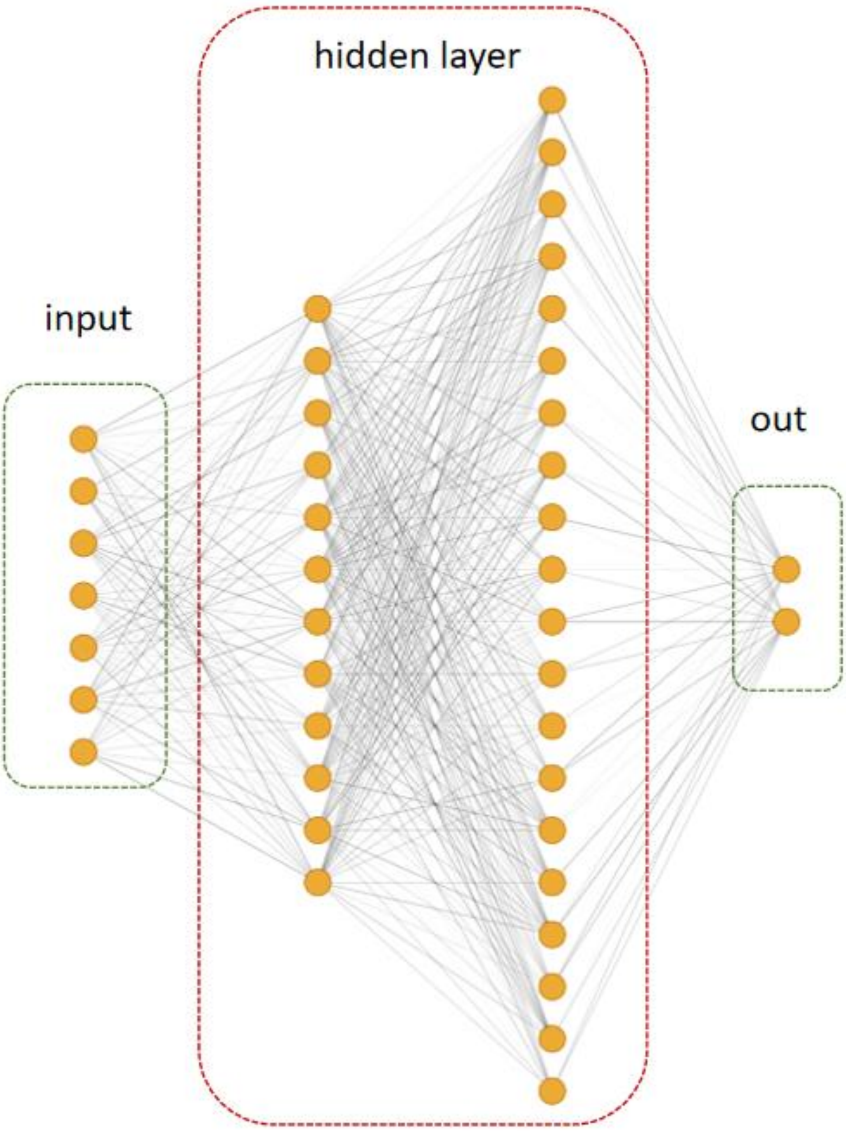

Figure3: Model structure.

2. Add activation function:

For the nonlinear expression ability of the model, we introduced the ReLU activation function to improve the accuracy and generalization ability of the model (Figure 4).

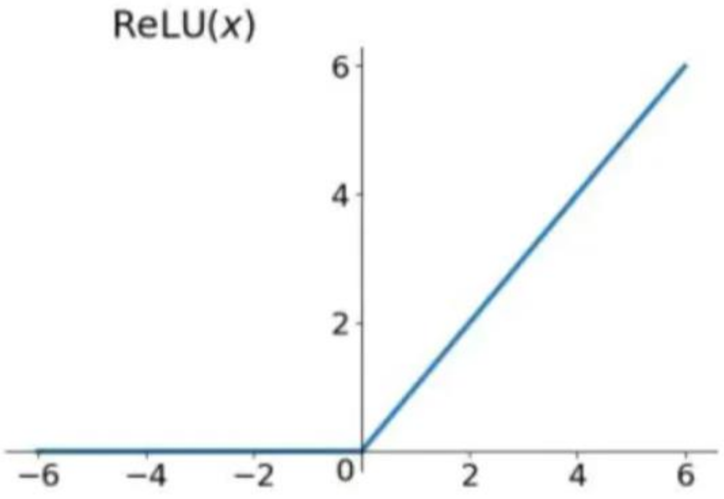

Figure4： ReLU activation function

3. Introduce Dropout:

In order to further prevent the model from overfitting, we added the Dropout method to the hidden layer to inactivate neurons with a certain probability, thereby reducing the probability of model overfitting. In order to search for a suitable dropout probability, we use this probability as a hyperparameter and use it in conjunction with the hyperparameter in the hidden layer to perform experiments. The experiment code is as follows:

```
channels = [
        (7, 16, 32, 2),
        (7, 32, 64, 2),
        (7, 64, 128, 2),
        (7, 128, 256, 2),
    ]
    dropout = [i / 10 for i in range(8)]
    for channel in channels:
        for drop in dropout:
            train(channel, drop)
```

The model structure construction code is as follows：

```
class DNNModel(nn.Module):
    def __init__(self, channels=(3, 64, 128, 2), dropout=0.5):
        super(DNNModel, self).__init__()
        self.net = nn.Sequential(
            nn.Linear(channels[0], channels[1]),
            nn.Dropout(p=dropout),
            nn.ReLU(),
            nn.Linear(channels[1], channels[2]),
            nn.Dropout(p=dropout),
            nn.ReLU(),
            nn.Linear(channels[2], channels[3])
        )
        initialize_model(self.net)
```

```
def forward(self, x):
    x = self.net(x.float())
    return x

def predict(self, x):
    x = F.softmax(self.forward(x), dim=1)
    return x
```

We record the loss of the model on the training set and validation set during the experiment, as shown in Figure 5:

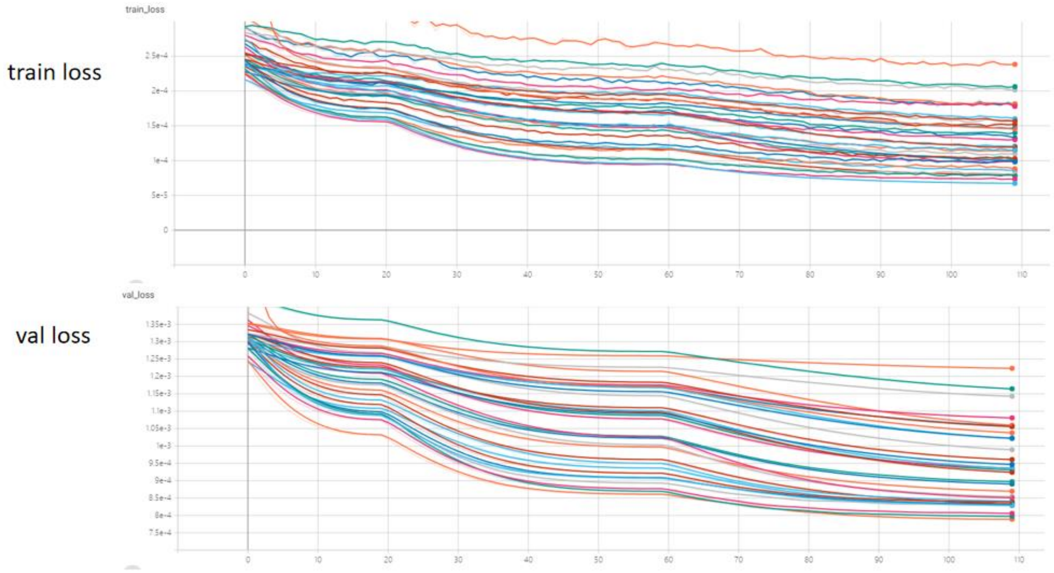

Figure5: The loss trend of the model in the training set and the validation set

It can be seen that the loss of the model on the training set and the validation set is in a steady downward trend, and there is no over-fitting phenomenon (over-fitting is generally manifested as the loss of the training set decreases, and the loss of the validation set no longer decreases or increases). At the same time, we choose to save the model at the minimum loss point on the validation set, which further prevents overfitting of the saved model.
